# Effects of Storage Time and Pre-Etching Treatment of CR-39 Detectors on Their Response to Alpha Radiation Exposure

**DOI:** 10.3390/ijerph18168346

**Published:** 2021-08-06

**Authors:** Miroslaw Janik, Md. Mahamudul Hasan, Peter Bossew, Norbert Kavasi

**Affiliations:** 1 Department of Radiation Measurement and Dose Assessment, National Institute of Radiological Sciences (QST/NIRS), Chiba 263-8555, Japan; janik.miroslaw@qst.go.jp; 2 Department of Environment Systems, Graduate School of Frontier Sciences, Kashiwa Campus, The University of Tokyo, Chiba 277-8561, Japan; mahamudullpe.du@yahoo.com; 3 Section Radon and NORM, German Federal Office for Radiation Protection (BfS), 10318 Berlin, Germany; pbossew@bfs.de; 4 Department of Environmental Sciences, Jožef Stefan Institute, 1000 Ljubljana, Slovenia

**Keywords:** radon, CR-39, ageing, sensitization, track density

## Abstract

Radon passive monitors based on solid state nuclear track detectors (SSNTD), especially CR-39, are widely used in radon and thoron studies. They may be subjected to the influence of external factors, like changing of temperature, humidity, and pressure, both before and during the measurement. Evaluation of the exposed detectors involves chemical processing, whose conditions also influence the measurement results. The aim of this study was to check several factors, as to whether they may modify the response of CR-39 detector: concerning the phase before evaluation, storage time, and temperature during storage; and concerning the evaluation procedure, etching time, and pre-etching treatment using hot water and carbon dioxide atmosphere. Two experiments were conducted by irradiation of CR-39 detectors using alpha particles emitted from a mono-energetic ^241^Am source and exposed in radon atmosphere. Track density dependence of the age of production was found to be statistically not significant. On the other hand, pre-etching treatment using hot water and carbon dioxide with different etching times showed statistically significant effects on track area, track sensitivity, and roundness. It was concluded that there are simple methods to increase performance of nuclear track detectors, and that storage time is not a factor of concern.

## 1. Introduction

Since the discovery of radon (^222^Rn) by Friedrich Ernst Dorn, a German chemist, in 1900, many measurement methods and techniques have been developed for it. Researchers are interested not only in measuring ^222^Rn gas itself, but also its other isotopes, such as ^220^Rn (known as thoron) decay products because of their greater contributions to dose that comes from natural background radiation in some high background radiation areas (HBRA) [1]. 

These days, the numbers of laboratories dealing with radon and thoron study are rising, because the radiological problem of Rn exposure is increasingly being taken seriously, as reflected by governments setting stricter Rn regulations [2]. One task in Rn mitigation policy is surveying of dwellings and workplaces. Laboratories are usually equipped with a low resolution system for track reading and analysis, based on an optical microscope, simple camera, scanner, and open-source software [3,4]. (This may be different for companies whose business is dealing with large-scale providing and processing of Rn detectors).

A variety of methods and instruments has been developed using different principles of detection for different measurement applications and, in general, the instruments can be divided into two main sampling method categories: active or passive [5]. Regarding the purposes for which they are commonly used, active devices can record time-series data in their memory for further analysis whereas passive registers generate values of time-integrated exposure or concentration, which renders them suitable for assessing long-term mean concentrations, as needed in Rn surveys. Most of the measurement techniques are based on detecting and recording of alpha particles emitted in the uranium decay chain from ^222^Rn, ^218^Po, and ^214^Po as well as in the thorium chain from ^220^Rn, ^216^Po, ^212^Bi, and ^212^Po. It should be mentioned that some measurement methods utilizing activated carbon provide analyses with HPGe or NaI spectrometers taking advantage of the distinct gamma ray energies of Rn decay products (^214^Pb, ^214^Bi). Other types of devices widely used in radon study are the electret ion chambers (EIC) wherein the electret (permanently charged Teflon disk) serves both as a source of electrostatic field and as a sensor. The ions produced inside the chamber are collected onto the electret causing a reduction of the surface charge on the electret. The reduction in charge is a measure of the ionization integrated over a period of exposure to alpha particles emitted by the decay process of radon gas and its decay products [6].

The monitors based on etched-track detectors are the most frequently used devices for long-term (from a few weeks to one year) radon and thoron measurements and for determination of the annual exposure of the general public to indoor radon for three reasons: cost per exposure, ease of transport, and ease of use by the end user. Their detection principle is based on the registration of alpha particle tracks in solid state materials. The most commonly used plastic materials for SSNTDs are cellulose nitrate (commercial name: LR 115) and allyl diglycol carbonate (CR-39). After exposure, the tracks are made visible by chemically or electrochemically etching, usually in NaOH or KOH solution with suitable normality at a proper temperature and proper etching time. Later, the tracks are counted by using a microscope connected to a photo camera and PC or by an automatic counting system. The density is calculated based on the number of tracks and the scanned area. Finally, the integrated radon and thoron concentration is determined using exposure time and the calibration factor which is provided by the SSNTD manufacturer or obtained during a calibration process.

A particular topic of concern has been storage time of SSNTDs, because in many cases, the detectors intended to be used in radon surveys have to be bought or prepared in bulk and also their evaluation is often not possible at once because of limited laboratory capacity; hence for logistic reasons, rather long storage times may occur. A number of studies conducted about the subject pointed out the contribution of ageing (storage in air before exposure) and fading (after exposure) to changing properties of CR-39 detectors for alpha particles [7,8,9,10]. Hardcastle and Miles reported that exposure of CR-39 for 12 months reduced the CR-39 based detector efficiency (the number of tracks over a given area for a giving radon exposure) by 23%, taking into account both ageing and fading [7]. Enomoto and Ishigure examined ageing and fading effects after exposure by ^252^Cf against storage temperature (from −80 °C to 35 °C) for one year [9]. They did not observe changes of track diameters within one year when CR-39 detectors were stored at −80 °C and −23 °C, whereas for their storage at other temperatures, the track diameters of α-particles decreased with the storage period, but there was also dependence on the temperature. Additionally, Enomoto and Ishigure found that the storage at the temperatures of −80 °C, −23 °C, and 4 °C for one year did not bring about any significant changes to the detection efficiency (number of tracks) but at 23 °C, the detection efficiency decreased by 8% in the one-year storage [9]. 

Regarding enhancement of detector performance, a number of experiments tested factors for improving CR-39 track sensitivity, like pre-etching treatment in CO_2_ atmosphere, ultraviolet (UV) irradiation, and combination of UV with CO_2_ as well as hot water [11,12,13,14]. Although the fundamental mechanism of the CR-39 track sensitivity enhancement of the CO_2_ treatment is not yet fully understood, several experiments demonstrated and confirmed this effect [15,16]. 

The effect of sensitization of CR-39 by post-irradiation treatment in CO_2_ atmosphere was first reported by Fujii et al. [17]. Though their research concerned mainly then-newly developed SR-90 detector, they found that ageing latent tracks in 2.5 bar CO_2_ atmosphere for 22 days increased the track sensitivity of CR-39 by a factor of about 1.5 for 6 MeV alpha particles. The effect of increasing track sensitivity using hot water (70 °C) was studied by Ichinose et al. [18]. They found that for a short time period, the pre-soaking process increased the track sensitivity until a certain time and then it dropped. The effect of hot water post-irradiation treatment was checked by Bartok and Csige [19]. They calculated track sensitivity using the equation: S =, where A =, *d* is track diameter, and *h* is thickness of the layer removed during etching. However, this formula is valid only for round tracks. In their study the track sensitivity of CR-39 detectors (TASTRACK, TASL, and UK) was increased by 40% after 60 min treatment in 100 °C of hot water. 

The current study presents the results of research for the CR-39 detector on the ageing effect, changes in track sensitivity, density and track geometry (diameter, area, and roundness) by examining the factors of storage time, storage temperature, pre-etching treatment, and etching time with hot water and CO_2_ gas, without considering the physical and chemical processes leading to track formation and growth.

This research investigated: (1) whether or not SSNTD detection efficiency was related to storage time even for more than 10 years; and (2) how pre-etching treatment of CR-39 in hot water and CO_2_ changed the track sensitivity, density, and other track parameters of this material. Details on the dependence of detection efficiency and track parameters on the treatment (etching) time and on the source of irradiation and exposure were also identified. Additionally, the experiments looked at how the treatments before etching affected the density of registered tracks (i.e., the performance values of track sensitivity or detector efficiency). 

## 2. Materials and Methods

The aim of this work was to show that even low-cost pre-etching treatment methods can significantly improve the track sensitivity of SSNTDs, which in turn may translate into better quality of tracks by customization of etching and image analyser processes. Improving the visibility of the microscopic image of the track in order to increase the track size and the image contrast between tracks will make the tracks easily distinguishable when, for example, detectors are exposed in a mixed radiation field, e.g., neutron and radon as presented by Hulber and Selmeczi [20]. 

The following parameters, bulk etch rate *V_b_*, track etch rate *Vt*, major and minor track diameters, track dimensions, area, and roundness characterize the etching process, track formation, and SSNTD physical parameters for a specific particle energy. A charged particle (for example, an α-particle emitted from an ^241^Am source) passing through a nuclear track detector leaves a radiation damage trail (called a latent track) along its trajectory. The etchant is removed preferentially along the latent track at the track etch rate (*V_t_*), while it is also removed from the surface generally at the bulk etch rate (*V_b_*). As a result, a conical etched track appears in the detector. A track is formed when *V_t_* > *V_b_*. Tracks obtained under an oblique incident angle can be used for determination of *V_t_*. To estimate the incident angle, the major and minor axes of the track openings are used. The dependence of *V_t_*/*V_b_* in CR-39 was examined in several experiments [21]. 

The detector sensitivity *S* (*S* = *V* – 1 = *V_t_*/*V_b_* − 1), can be used to evaluate the effect of etching conditions. The *S* function as a function of track parameters can be expressed as presented by Equation (1) [22].

(1) $$S=V-1=-1=-1;\;R=\frac{D}{2\;h};\;r=\frac{d}{2\;h}$$

where *R* and *r* are the major and minor radius of the track opening expressed in µm after etching removed layer *h* and *D* and *d* are the major and minor diameters of the track, respectively. 

The decision on whether a particle is detected or not (i.e., whether it will produce a visible track after etching) also differs between researchers. In some papers, the main criterion is the critical angle. A particle will be detected only if it strikes the detector above the critical angle. However, the critical angle is a function of the particle energy and the etching conditions. Furthermore, this function is rather complicated and no single value for the critical angle can be assumed but it has been found that chemical etching conditions are among the main factors affecting the relationship between critical angle and incident energy [23]. 

Roundness is defined as N = (*D* and *d* are the major and minor axes track diameter) and it can be related to incident angle (δ) and critical angle but the relationship of N(δ) is not discussed here. However, this parameter is also useful for optimizing the etching and reading processes to improve the visibility of the track.

In this study, CR-39 detectors were used; they are commercially known as BARYOTRAK (Fukuvi Chemical Industry, Japan), and they are sold as a neutron personal dosimetry product. BARYOTRAK is made from 99.7% purified monomer and provides a uniform and clean surface without any distortions or bubble-like noise caused by etching damage [24]. 

### 2.1. Experiment 1: Ageing Effect and Track Density (at Refrigerator and Room Temperatures)

In the present study, two levels of storage temperature were tested: (1) storage in a refrigerator with a stable temperature of 7.3 °C; and (2) storage at room temperatures varying from monthly averages of 13.8 °C in mid-winter to 25.9 °C in mid-summer.

A set (S1 to S18) of detectors was irradiated for 3 min by a ^241^Am source with activity of 500 Bq. Pre-etching treatment consisted of keeping the exposed detectors in 1 L hot water at 98 °C for 1 h (HW treatment) or keeping it in a commercially available soda syphon in which the gas pressure was 18 g/L CO_2_ (~0.87 MPa) for 66 h (CO_2_ treatment). The chemical etching (NaOH, 6 M, 70 °C, 24 h) was started as soon as possible after pre-etching treatment. In each subgroup, denoted in Table 1 as e.g., S1-T1-NO, 10 detectors were exposed to allow reasonable statistical evaluation, while one was stored and evaluated under the same conditions as irradiated detectors, to determine the background. In total 600 SSNTD were exposed. All treatment conditions are summarized in Table 1. Etched tracks were counted manually by viewing through a microscope.

**Figure 1:**
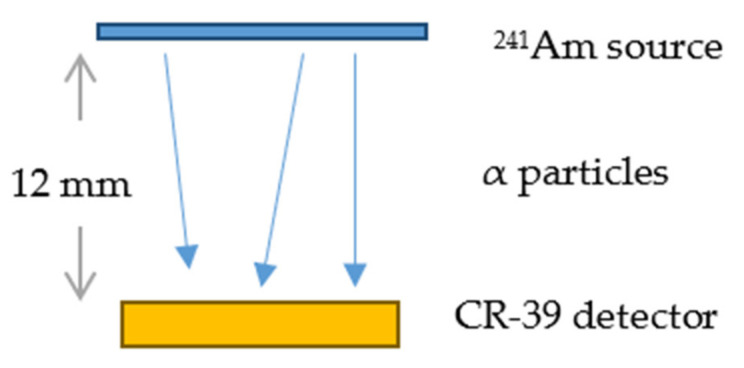
Schematic of ^241^Am irradiation.

A sketch of the exposure set-up is presented later in Figure 1.

### 2.2. Experiment 2: Evaluation of Etched-Track Properties

**Figure 2:**
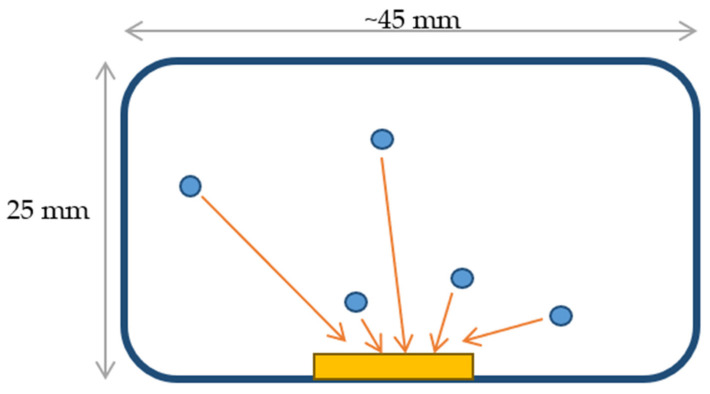
Schematic view of radon (right) exposure [27].

The objective of Experiment 2 was to check if the etching time and treatment conditions had any influence on the track properties, i.e., track density, track diameters [µm], track area (etched track) [µm²], and roundness [–]. For this purpose, five sets of detectors (SB1 to SB5) were irradiated by an ^241^Am source with activity of 1000 Bq for 3 min. Moreover, in order to study the response of the CR-39 in a realistic environment the experiment using a radon exposure system consisting of a 150 L steel chamber, a radon source and an environmental control system was carried out [25]. In this study, one part (the low-diffusion chamber) of the radon-thoron discriminative passive monitor, known as the RADUET, was used. The RADUET is composed of two diffusion chambers with different air exchange rates in each of which a CR-39 detector is deployed. Details about investigation of the effects of the different air exchange properties of the RADUET can be found in [26,27]. Schematics of the RADUET and irradiation of a mounted CR-39 detector by alpha particles emitted by radon and its progeny are shown in Figure 2. The experiment using the radon chamber system had exposure of 60 RADUETs, which were divided into five groups (SC1–SC5). Since all RADUETs were exposed at the same time, the number of monitors was limited by the volume of the radon chamber. After exposure, the detectors were removed from the low-diffusion chamber, in which thoron does almost not contribute. 

Each group of monitors, i.e., SB1–SB5 and SC1–SC5 (Table 2) was divided into subgroups (four exposed detectors and one background detector per subgroup) treated differently before etching, i.e., no-pre-treatment (NO), hot water treatment (HW), and CO_2_ treatment (CO_2_). After irradiation or exposure NO and HW subgroups were stored in a refrigerator in a radon-proof bag, whereas the CO_2_ subgroup was kept in 18 g/L CO_2_ (~0.87 MPa) for 66 h. HW detectors were preserved in hot water (98 °C) for 1 h just before etching. In order to maximize the pre-etching treatment effect, the time between finishing the pre-etching treatment and etching in NaOH was as short as possible, i.e., less than 10 min. Then, etching of CR-39 detectors started simultaneously and was finished in the scheduled times, i.e., 4.5, 9, 13.5, 18, and 24 h. Finally, the counting systems for reading and analysis described elsewhere were applied [28]. 

### 2.3. Statistical Evaluation

The effects of storage time, storage temperature, and etching time on the dependent variables were assessed by analysis of variance (ANOVA). The null hypothesis for ANOVA is that the arithmetic mean (average value of the dependent variable) is the same for all groups. The alternative hypothesis is that the mean is not the same for all groups. The way of deciding is a comparison of variances of the response variable between and within groups. The criterion for rejecting the null hypothesis is exceedance of a critical value of a certain statistic (F). The F value in ANOVA is therefore a tool to help answer the question, “Are the means of two populations significantly different?” In the test, F also determines the *p*-value; the *p*-value is the probability the difference is at least as high as the one that was actually observed, given that the null hypothesis is true. Statistical significance was accepted at a *p* below a chosen significance level, α; here it was assigned as 0.05. 

In analogy, two-way ANOVA is used to estimate how the mean of a quantitative response (or dependent) variable changes according to the levels of two categorical grouping variables, also called factors. Two-way ANOVA was judged appropriate to this study because it enables analysis of how two independent variables (e.g., storage time and temperature) affect a dependent variable (track density). In addition, two-way ANOVA includes investigating interaction between the two grouping variables, since it may be that grouping of the response variable by one grouping variable is different according to the level of the second one. Whenever this is the case, statistical significance is again assessed through a *p*-value. If it is not the case, grouping by the two categorical variables can be investigated independently. Three-way ANOVA proceeds in analogy.

The analyses and figures were carried out using R-software with additional libraries [29,30]. The results are given in tables which report as outputs the *p*-value (*p*) which serves for deciding about significance of whether grouping is present.

## 3. Results and Discussion

### 3.1. Experiment 1: Ageing Effect and Track Density (at Refrigerator and Room Temperatures)

The ageing effect was checked for 18 sets of NO subgroup detectors (S1–S18), 12 HW subgroup detectors (S1, S2, S4, S6, S9, and S12–S18) and six CO_2_ subgroup detectors (S1, S2, S4, S6, S9, and S12). However, the initial evaluation showed a problem with S6–T1–NO data without any recognizable physical reason, therefore this set was regarded as an outlier and excluded from further consideration. Storage temperature effect was tested when detectors were kept at both refrigerator and room temperatures. 

**Figure 3:**
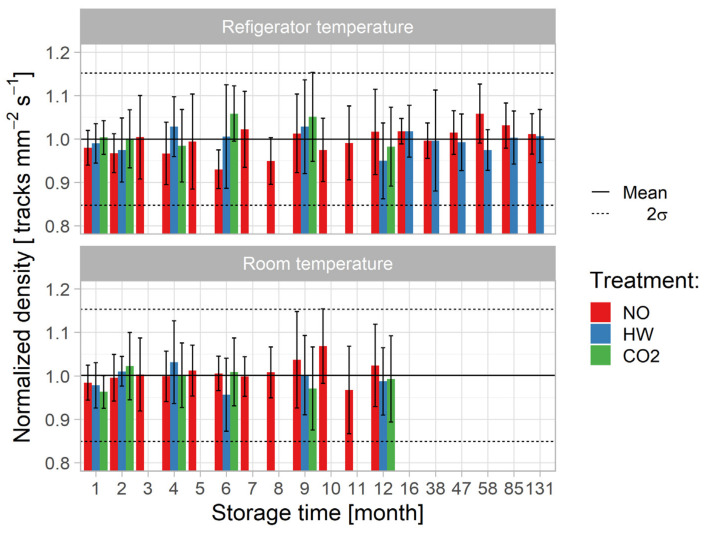
Track density versus storage time and temperature (the results were normalized by the irradiation time since different irradiation times were used, ranging from 180–190 s).

In the first step of analysis, three-way ANOVA analysis with interaction was carried out under the assumption that all pre-etching treatments were performed for both storage temperatures. This postulate was validated for five periods, i.e., 1-, 2-, 4-, 9-, and 12-month periods, as shown in Figure 3.

The results presented in Table 3 suggested that the null hypothesis *(p < 0.05)* should not be rejected as there are no statistical differences in the mean track density.

The detailed analysis for each pre-etching treatment against storage time (ST) and storage temperature (TE) was conducted using two-way ANOVA analysis and these results are presented in Table 4. The number of detectors for ST effect was different between NO and the other (HW and CO_2_) pre-etching treatments because of the different numbers of experiments performed: NO was performed for S1–S5 and S7–S12 subgroup, whereas HW and CO_2_ were accomplished for S1, S2, S4, S6, S9, and S12 subgroups. Again, results did not show statistical significance. 

The effects of pre-etching treatment (PE) and storage temperature (TE) in the monthly periods were analysed as presented in Table 5. Five subgroups (S1, S2, S4, S9, and S12) for all three (NO, HW, and CO_2_) pre-etching treatments were taken into consideration. No statistical significance was found.

The effects of storage time (ST) and pre-etching treatment (PE) against storage temperature (TE) was analysed using two-way ANOVA and results are shown in Table 6. At refrigerator temperature, it was possible to distinguish between storage time when all (NO, HW, and CO_2_) pre-etching treatments were conducted at months 1, 2, 4, 9, and 12 and only two pre-etching conditions (NO and HW) at months 1, 2, 4, 9, 12, and 16. No statistical significance was found. 

Finally, one-way ANOVA was performed: to check the storage temperature (TE) for each factor, storage time (ST), and pre-etching treatment (PE) (Table 7); to check the storage time (ST) for each storage temperature (TE) and pre-etching treatment (PE) (Table 8); and to check the pre-etching treatment (PE) effect for each storage time (ST) and storage temperature (TE) (Table 9). 

It was observed that storage temperature had statistical significance for three cases (8–NO, 10–NO and 1–CO_2_). No statistical significance was observed for storage time at each of the storage temperatures and each of the pre-etching treatments. An effect from the pre-etching was seen only for one case (58–Refrigerator temperature) when NO and HW pre-etching treatments were applied. 

### 3.2. Experiment 2: Evaluation of Etched-Track Properties

Experiment 2 using an irradiation by ^241^Am source and exposure in the radon chamber was intended for analysis of the track density, track size (diameter and area), and sensitivity with different pre-etching treatments and different etching times.

#### 3.2.1. ^241^Am Exposure

**Figure 4:**
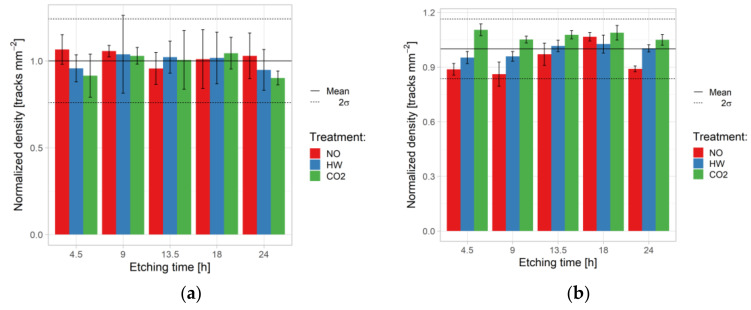
Track density vs. etching time for three pre-etching treatments in Experiment 2: ^241^Am irradiation (**a**) and radon (**b**) exposures.

Track density for each etching time and pre-etching condition is presented in Figure 4a. It can be observed that the density differences for each exposure are not higher than 2σ (i.e., 13%) and follow results from Experiment 1. 

**Figure 5:**
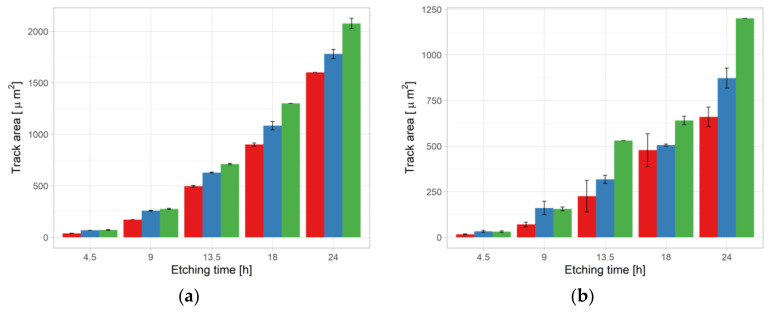
Mean track area vs. etching time for three pre-etching treatments in Experiment 2:^241^Am irradiation (**a**) and radon exposure (**b**). For color codes see Figure 3 and Figure 4.

Figure 5a shows the track sizes difference obtained in the ^241^Am exposure of Experiment 2. It was evident that both etching time and pre-etching treatments have an influence on track size with the sequence NO < HW < CO_2_. 

**Figure 6:**
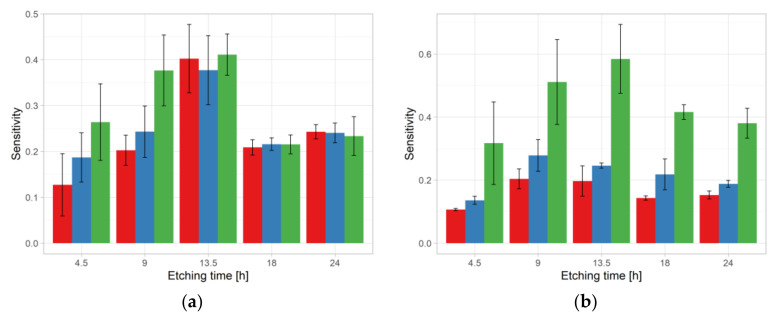
Sensitivity results obtained in Experiment 2 for ^241^Am irradiation (**a**) and radon exposure (**b**). For color codes see Figure 3 and Figure 4.

Sensitivity results are shown in Figure 6a. Increasing of sensitivity was observed in the sequence NO < HW < CO_2_ for 4.5 and 9 h etching time. With an etching time of 13.5 h, on the one hand, the sensitivity showed the highest values and, on the other hand, practically did not differ for NO, HW, and CO_2_. For the longest two etching times, i.e., 18 and 24 h, sensitivities were about 50% lower than those under for 13.5 h and values are similar between treatments. 

It should be mentioned that in the case of 4.5 h etching time the sensitivity for HW was almost 40% higher than for NO pre-treatment and was in good agreement with results presented in the other study [19] where CR–39 was etched for 5 h. 

**Figure 7:**
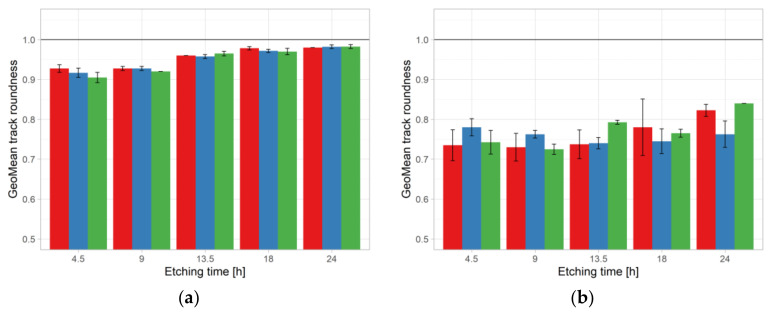
Roundness results obtained in Experiment 2 for ^241^Am irradiation (**a**) and radon exposure (**b**). For color codes see Figure 3 and Figure 4.

Table 10 summarizes the two-way ANOVA results for track area, roundness, and density against pre-etching treatment and etching time in Experiment 2 for ^241^Am exposure. In the case of ^241^Am, the effects of pre-etching treatment and etching time were statistically significant for track area and roundness. The interaction term was statistically significant and showed that both factors were needed to explain the overall effect (track area). For roundness, the interaction, although statistically significant, was difficult to interpret, as no clear trend could be recognized (Figure 7a). The effect on density is not statistically significant.

#### 3.2.2. Radon Exposure

The situation was somewhat different for the radon exposure. Table 11 shows that the effect of pre-etching treatment and etching time were statistically significant to track area and roundness, as for the ^241^Am parameters. On the other hand, in contrast to ^241^Am exposure, the analysis of track density also showed significance (Figure 4b). Regarding the track area, the impact of etching time was clearly visible (Figure 5b). Mean track area vs. etching time for three pre-etching treatments in Experiment 2:^241^Am irradiation (a) and radon exposure (b). For color codes see Figure 3 and Figure 4.

The sensitivity showed the same treatment effect sequence for all etching times, i.e., lowest for NO and highest for CO_2_ pre-etching condition (Figure 6b).

Regarding roundness, no effect could be recognized, but remarkably lower from the ^241^Am irradiation (Figure 7b). Finally, for track density the strongest effects were observed for CO_2_ treatment. 

Table 12 and Table 13 report the results of the one- and two-way ANOVA. The effects of etching temperature and pre-etching treatment are both significant, *p* < 0.01, except CO_2_ effect (*p* = 0.08) for density and 18h etching time for density (*p* = 0.13) also, and roundness (*p* = 0.14).

Effects on density and roundness are not statistically significant for each etching time. The one on area is statistically significant whereas the one on sensitivity, as described earlier, is statistically significant for the first two etching times (4.5 and 9 h). Pre-etching condition has a significant impact to results, except on density. For the rest of the checked parameters, i.e., area, roundness, and sensitivity, the effects were statistically significant. 

#### 3.2.3. Comparison of ^241^Am Irradiation and Radon Exposure Results and Physical Interpretation

**Figure 8:**
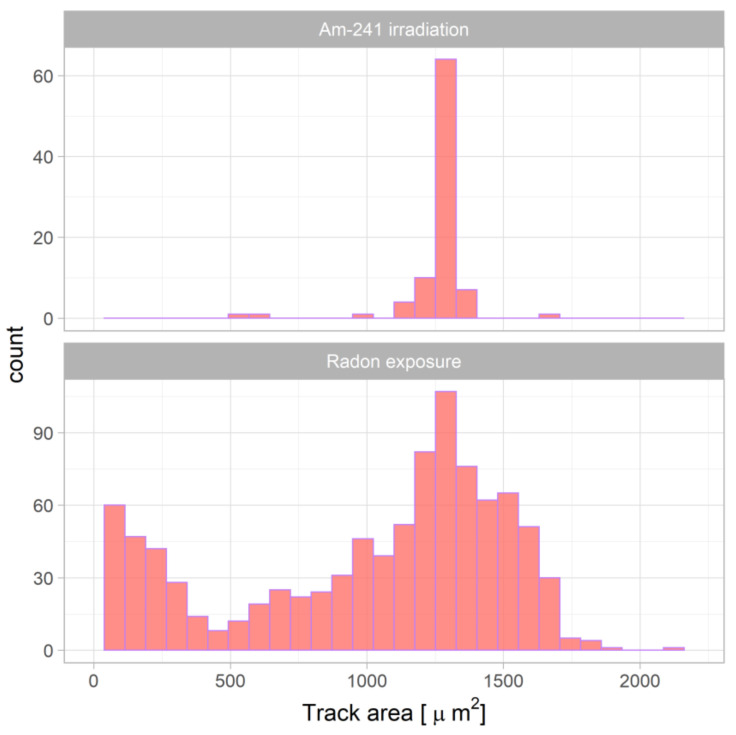
Spectra of track areas obtained in Experiment 2 for ^241^Am irradiation (upper) and radon exposure (lower).

It was observed that the mean track area was much lower for radon exposure than for ^241^Am irradiation. This was caused by the different track distribution as presented in Figure 8 (an example for CO_2_ pre-etching treatment with 24 h etching time). The difference could be explained by the difference of the energy of alpha particles reaching the CR-39. The peak of registered alpha particles from ^241^Am corresponds to the most probable energy of 4.4 MeV for 12 mm attenuation in air (distance from the source to CR-39 detector) calculated by SRIM software [31]. In the case of the radon exposure, alpha particles emitted from radon and its progeny had different energies and hit the CR-39 detector from different distances, limited by the detector size. Therefore, the registered energy range was much wider than that of mono-energy alpha particles emitted from the ^241^Am source. 

Apart from the track area, the roundness of tracks should be taken into consideration. The comparison of roundness results obtained in Experiment 2 from ^241^Am irradiation and radon exposure against etching time is presented in Figure 7. It was observed for ^241^Am irradiation that the difference between minimal and maximal values of roundness was 0.08, ranged from 0.9 (CO_2_ with 4.5 h etching time) to 0.98 (CO_2_ with 24 h etching time). The likely explanation for such a large value is that the angle of incidence was not far from 90°. The difference between maximal and minimal roundness of tracks obtained in the radon exposure was 0.18, ranged from 0.69 (CO_2_ treatment with 9 h etching time) to 0.87 (NO treatment with 24 h etching time). It seems that for radon exposure, roundness tended to be lower than that for ^241^Am irradiation. The wide range for radon exposure could be connected to the detector chamber design and, as said previously, to the distance from the source, i.e., alpha particles released in decay of radon and its progeny were originating in a volume rather than a plane source, with corresponding differently distributed angles of incident [32].

The track density analysis showed different results between both experiments, namely, no statistical significance for ^241^Am exposure and statistically significance for the radon experiment. This behaviour can be connected, as previously, to experiment design and alpha particle angle of incident. 

The track sensitivity of detectors was obtained from geometrical parameters of each detector using the formula of Equation (1). The tendency of sensitivities can be attributed to the physical nature of track formation and overetched tracks. In brief, during etching, the chemically aggressive solution (e.g., NaOH) progresses toward the end point of the particle trajectory. The track end is sharp and the track is fully conical. With prolonged etching, the spherical part of the track wall, and thus the circular part of the track opening, are enlarged. Finally, the track becomes totally spherical and the opening becomes completely circular, which indicates that the track is overetched. Detailed descriptions about the formation process of tracks, various models and their limitations can be found in [33]. Tracks tended to be overetched as presented in Table 14. with the etching time of more than 9 h, indicated by the fact that the centre of the track is not black. However, the difference in tendency between ^241^Am irradiation and Rn exposure points to some other currently unknown reason which we cannot explain at this moment.

## 4. Conclusions

The aims of this paper were to investigate the effects of storage of solid state nuclear track detector (SSNTD) before exposure and of their treatment before and during evaluation. Factors considered were storage time and temperature before exposure, etching time and pre-etching treatment. Response variables were detector density (factors: storage time, temperature, and pre-etching treatment; Experiment 1), track area, roundness, and density; Experiment 2). In addition, in Experiment 2, response differences between irradiation by ^241^Am and exposure to radon were investigated. 

First, the ageing effect was analysed for SSNTD CR-39 detectors irradiated using ^241^Am alpha source; detectors’ ages before the exposure ranged from one month to almost 10 years, starting from their date of manufacture. Detectors had been stored at two temperature conditions for one year. Fluctuation in track density was observed in the range of 8% but without statistically significant differences (ANOVA, *p* < 0.05). These results were in line with results presented by Calamosca and Penzo [10] who found that ageing and fading had small effects on CR-39 stored at ambient temperature. Cecchini et al. [34] also found no indications of ageing effects for material older than 10–12 months.

On the other hand, the present results were in disagreement with the conclusions of some investigators [35] who, however, used detectors made of another plastic material. For better and deeper explanation another type of experiment (mainly chemical and checking the interaction at the atomic level) should be accomplished in the future.

The present results showed that the track sensitivity was different, depending on pre-etching treatments; this is in agreement with other published results [12,13,14,19]. 

For differences of response between exposures to ^241^Am and radon, a tentative physical explanation could be given.

It was concluded that the results proved that the track densities for detectors kept in ambient conditions for up to 12 months did not affect the reliability of passive radon monitors when using CR-39 detectors. Furthermore, the presented experiments showed ways to improve detector performance through increasing track size (diameter and area) by utilization of low-cost, simple, and effective pre-etching treatments, i.e., hot water and low-pressure gas treatment using carbon dioxide generated in a soda syphon for ordinary household use.

When SSNTD CR-39 detectors are to be used for radon monitoring, the following recommendations were concluded to be important: (1) to check the track density for new and old CR-39 detectors due to different material compositions; and (2) to check the effects of pre-etching treatments when comparing or interpreting experimental results in different studies.

## Figures and Tables

**Table 1:** Treatment conditions for Experiment 1.

Set	S1		S2		S3		S4		S5		S6		S7		S8		S9		S10		S11		S12		S13	S14	S15	S16	S17	S18
Month	1		2		3		4		5		6		7		8		9		10		11		12		16	38	47	58	85	131
Temp	T1	T2	T1	T2	T1	T2	T1	T2	T1	T2	T1	T2	T1	T2	T1	T2	T1	T2	T1	T2	T1	T2	T1	T2	T1	T1	T1	T1	T1	T1
NO	X	X	X	X	X	X	X	X	X	X	X	X	X	X	X	X	X	X	X	X	X	X	X	X	X	X	X	X	X	X
HW	X	X	X	X			X	X			X	X					X	X					X	X	X	X	X	X	X	X
CO_2_	X	X	X	X			X	X			X	X					X	X					X	X						

Set, number of set; Month, months from CR-39 manufacture day. Temp, temperature treatment: T1, refrigerator temperature; T2, room temperature. Treatment conditions: NO, no pre-treatment detectors; HW, treatment in hot water; and CO_2_, treatment in CO_2_ atmosphere.

**Table 2:** Conditions for Experiment 2 (^241^Am irradiation and radon treatment).

Set	SB1/SC1	SB2/SC2	SB3/SC3	SB4/SC4	SB5/SC5
Etching time [h]	4.5	9	13.5	18	24
NO	X	X	X	X	X
HW	X	X	X	X	X
CO_2_	X	X	X	X	X

Set, number of set. Treatment conditions were the same as given in Table 1.

**Table 3:** Three-way ANOVA results of track density *.

Effect	*p*
ST	0.17
TE	0.62
PE	0.99
Interaction ST:TE	0.20
Interaction ST:PE	0.23
Interaction TE:PE	0.29
Interaction ST:TE:PE	0.87

* ST, storage time; TE, storage temperature; and PE, pre-etching treatment.

**Table 4:** Fixed pre-etching treatment—two-way ANOVA *.

PE	Effect	*p*
NO	ST	0.33
	TE	0.06
	Interaction ST:TE	0.47
HW	ST	0.17
	TE	0.92
	Interaction ST:TE	0.49
CO_2_	ST	0.17
	TE	0.92
	Interaction ST:TE	0.49

* ST, storage time; TE, storage temperature; and PE, pre-etching treatment.

**Table 5:** Fixed storage time—two-way ANOVA *.

ST	Effect	*p*
1	PE	0.99
	TE	0.16
	Interaction PE:TE	0.25
2	PE	0.30
	TE	0.07
	Interaction PE:TE	0.94
4	PE	0.13
	TE	0.38
	Interaction PE:TE	0.84
9	PE	0.90
	TE	0.29
	Interaction PE:TE	0.27
12	PE	0.20
	TE	0.43
	Interaction PE:TE	0.85

* ST, storage time; TE, storage temperature; and PE, pre-etching treatment.

**Table 6:** Fixed temperature—two-way ANOVA *.

TE	Effect	*p*
Refrigerator (NO, HW, and CO_2_)	ST	0.09
	PE	0.59
	Interaction ST:PE	0.37
Refrigerator (NO, HW)	ST	0.31
	PE	0.28
	Interaction ST:PE	0.09
Room (NO, HW, and CO_2_)	ST	0.45
	PE	0.51
	Interaction ST:PE	0.53

* ST, storage time; TE, storage temperature; and PE, pre-etching treatment.

**Table 7:** Storage temperature effect—one-way ANOVA *.

ST	PE	Effect	*p*
1	NO	TE	0.79
2	NO	TE	0.21
3	NO	TE	0.99
4	NO	TE	0.28
5	NO	TE	0.65
7	NO	TE	0.46
**8**	**NO**	TE	**0.03**
9	NO	TE	0.60
**10**	**NO**	**TE**	**0.02**
11	NO	TE	0.59
12	NO	TE	0.86
1	HW	TE	0.61
2	HW	TE	0.18
4	HW	TE	0.92
9	HW	TE	0.56
12	HW	TE	0.32
**1**	**CO_2_**	TE	**0.03**
2	CO_2_	TE	0.50
4	CO_2_	TE	0.64
6	CO_2_	TE	0.14
9	CO_2_	TE	0.09
12	CO_2_	TE	0.80

* ST, storage time; TE, storage temperature; and PE, pre-etching treatment; bold format, *p* < 0.05.

**Table 8:** Storage time effect—one-way ANOVA *.

TE	PE	Effect	*p*
T1	HW	ST	0.60
T1	CO_2_	ST	0.12
T2	NO	ST	0.27
T2	HW	ST	0.32
T2	CO_2_	ST	0.55

* ST, storage time; TE, storage temperature; and PE, pre-etching treatment.

**Table 9:** Pre-etching treatment effect—one-way ANOVA.

ST	TE	Effect	*p*
1	T1	PE	0.45
2	T1	PE	0.48
4	T1	PE	0.19
9	T1	PE	0.70
12	T1	PE	0.28
16	T1	PE	0.99
38	T1	PE	0.99
47	T1	PE	0.40
**58**	**T1**	**PE**	**<0.01**
85	T1	PE	0.29
131	T1	PE	0.85
1	T2	PE	0.55
2	T2	PE	0.60
4	T2	PE	0.58
6	T2	PE	0.19
9	T2	PE	0.35
12	T2	PE	0.63

ST, storage time; TE, storage temperature; and PE, pre-etching treatment; bold format, *p* < 0.05.

**Table 10:** ANOVA results for the ^241^Am exposure in Experiment 2 *.

Source	*p*
Track area	
PE	**<0.01**
ET	**<0.01**
Interaction PE:ET	**<0.01**
Roundness	
PE	**<0.01**
ET	**<0.01**
Interaction PE:ET	**<0.01**
Density	
PE	0.52
ET	0.51
Interaction PE:ET	0.76
Sensitivity	
PE	**<0.01**
ET	**<0.01**
Interaction PE:ET	**<0.01**

* ET, etching time; and PE, pre-etching treatment; bold format, *p* < 0.05.

**Table 11:** ANOVA results for radon exposure in Experiment 2 *.

Effect	*p*
Track area	
PE	**<0.01**
ET	**<0.01**
Interaction PE:ET	**<0.01**
Roundness	
PE	**<0.01**
ET	**<0.01**
Interaction PE:ET	**<0.01**
Density	
PE	**<0.01**
ET	**<0.01**
Interaction PE:ET	**<0.01**
Sensitivity	
PE	0.00
ET	0.00
Interaction PE:ET	0.14

* ET, etching time; and PE, pre-etching treatment; bold format, *p* < 0.05.

**Table 12:** One-way ANOVA for pre-etching effect with fixed etching time.

	4.5	9.0	13.5	18	24
Density					
^241^Am	0.154	0.958	0.743	0.933	0.272
Radon	**<0.01**	<0.05	0.02	0.13	**<0.01**
Area					
^241^Am	**<0.01**	**<0.01**	**<0.01**	**<0.01**	**<0.01**
Radon	**<0.01**	**<0.01**	**<0.01**	**<0.01**	**<0.01**
Roundness					
^241^Am	0.06	0.04	0.1	0.053	0.645
Radon	**<0.01**	**<0.01**	0.01	0.14	**<0.01**
Sensitivity					
^241^Am	0.07	0.005	0.762	0.741	0.889
Radon	**<0.01**	**<0.01**	**<0.01**	**<0.01**	**<0.01**

Bold format, *p* < 0.05.

**Table 13:** One-way ANOVA of track density for etching time effect with fixed pre-etching condition.

	NO	HW	CO_2_
Density			
^241^Am	0.715	0.848	0.236
Radon	**<0.01**	0.02	0.08
Area			
^241^Am	**<0.01**	**<0.01**	**<0.01**
Radon	**<0.01**	**<0.01**	**<0.01**
Roundness			
^241^Am	**<0.01**	**<0.01**	**<0.01**
Radon	**<0.01**	**<0.01**	**<0.01**
Sensitivity			
^241^Am	**<0.01**	**<0.01**	**<0.01**
Radon	**<0.01**	**<0.01**	0.01

Bold format, *p* < 0.05.

**Table 14:** Tracks obtained for different etching times and pre-etching treatments for ^241^Am irradiation.

	ET	4.5 h	9 h	13.5 h	18 h	24 h
PE						
**NO**		* 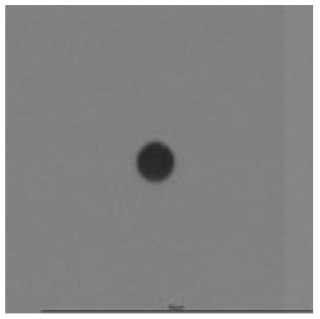 *	* 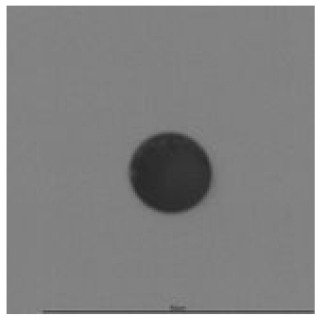 *	* 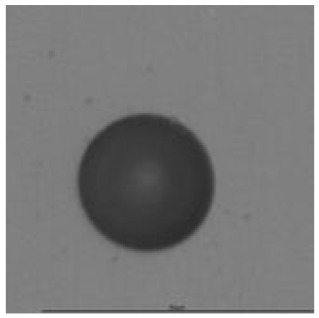 *	* 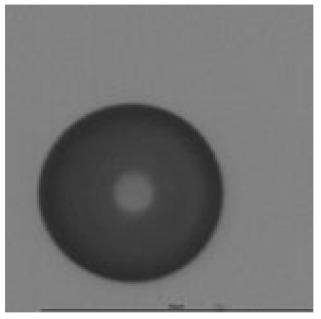 *	* 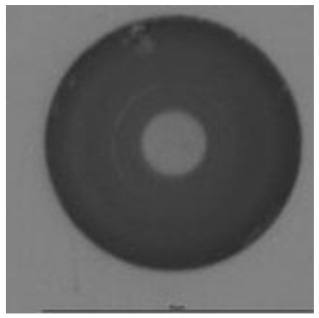 *
**HW**		* 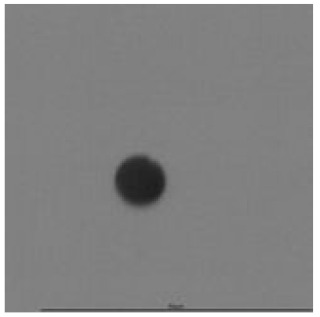 *	* 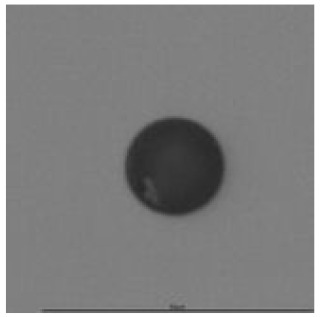 *	* 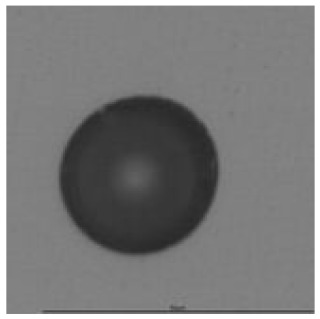 *	* 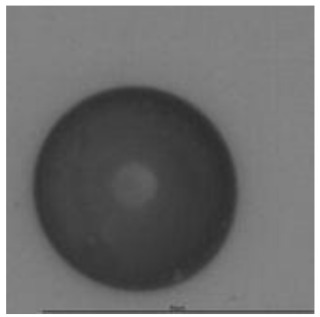 *	* 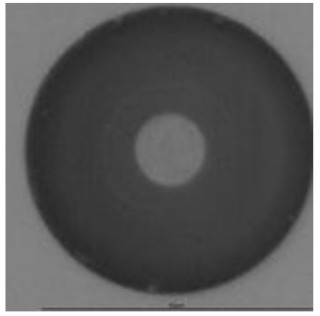 *
**CO_2_**		* 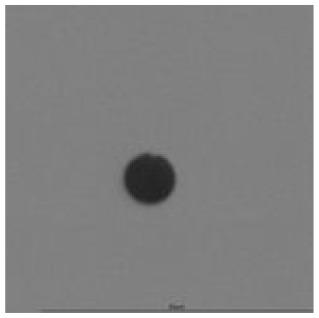 *	* 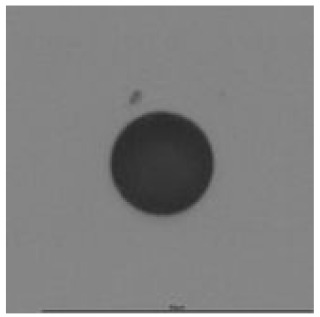 *	* 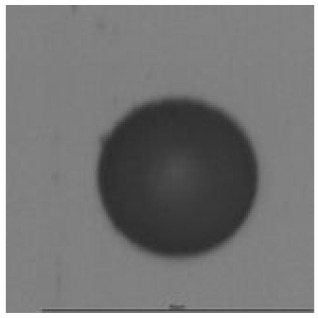 *	* 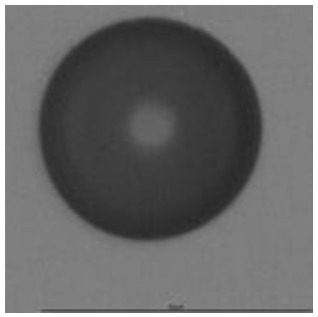 *	* 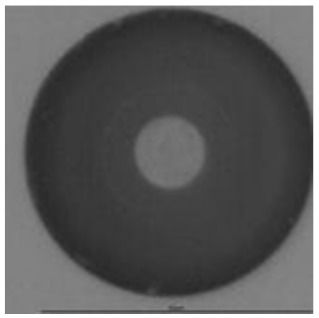 *

## Data Availability

The data presented in this study are available from the corresponding author on reasonable request.
